# Post-marketing safety of pentosan polysulfate sodium: a 21-year pharmacovigilance analysis of the FAERS database

**DOI:** 10.3389/fmed.2025.1725094

**Published:** 2026-01-22

**Authors:** Ben Wang, Long Xia, Er-hao Bao, Yong-bo Zheng, Yu-han Li, Dan Han, Tian-yi Shao, Xian-zhi Liu, Ping-yu Zhu

**Affiliations:** 1 Department of Urology, Affiliated Hospital of North Sichuan Medical College, Nanchong, Sichuan, China; 2 Department of Urology, Sichuan Provincial People's Hospital East Sichuan Hospital & Dazhou First People's Hospital, Dazhou, China; 3 Department of Ultrasound, Affiliated Hospital of North Sichuan Medical College, Nanchong, China

**Keywords:** adverse drug events, FAERS database, maculopathy, pentosan polysulfate sodium, pharmacovigilance

## Abstract

**Objective:**

To characterize the post-marketing safety profile of pentosan polysulfate sodium (PPS) using the FDA Adverse Event Reporting System (FAERS) database.

**Methods:**

This pharmacovigilance study analyzed FAERS data spanning from the first quarter of 2004 to the first quarter of 2025. Disproportionality analysis, using the reporting odds ratio (ROR), was performed to detect adverse drug event (ADE) signals. A gender-specific analysis was also performed. The time-to-onset (TTO) of ADEs was evaluated through descriptive statistics, the Kaplan–Meier method, and Weibull parametric modeling.

**Results:**

The study collected 11,471 reports with PPS as the primary suspected drug. The reporting frequency and strongest signals were overwhelmingly concentrated in the ‘Eye Disorders’ system organ class (SOC), with pigmentary maculopathy demonstrating an exceptionally high ROR. Significant non-ocular signals were also identified, including depression and anxiety. A gender-specific analysis revealed that maculopathy signals were prominently observed among females, while males exhibited distinct associations with gastrointestinal and urinary adverse events. The TTO analysis (*n* = 297) revealed a median onset time of 1,715 days, with the Weibull model (*β* = 0.62) indicating a decreasing hazard rate over time. The majority of reported cases (68.1%) were classified as serious adverse events.

**Conclusion:**

This 21-year real-world analysis confirms that safety signals for PPS show a distinct long-latency risk profile, most critically vision-threatening maculopathy. The study highlights potential psychiatric adverse events not currently documented in the drug’s label and reveals gender-specific risk profiles. These findings emphasize the need for vigilant, sustained ophthalmologic screening and broader safety monitoring for all patients receiving PPS therapy.

## 1 Introduction

Interstitial cystitis/bladder pain syndrome (IC/BPS) is a chronic and debilitating clinical condition characterized by suprapubic pain related to bladder filling, often associated with other symptoms such as urinary frequency and urgency, in the absence of a urinary tract infection or other obvious pathology (1). The condition mostly affects women and significantly impairs quality of life due to its chronic nature and difficulties in management (2). The etiology of IC/BPS is supposed to be multifactorial, with proposed mechanisms including urothelial dysfunction, mast cell activation, and neurogenic inflammation (3). A key hypothesis suggests that a compromised glycosaminoglycan (GAG) layer on the bladder urothelium permits urinary irritants to penetrate the bladder wall, initiating inflammatory cascade and sensory responses (4).

Pentosan polysulfate sodium (PPS), a synthetic sulfated polysaccharide, was approved by the U.S. Food and Drug Administration (FDA) on September 26, 1996, and continues to be the only oral medication especially for treating IC/BPS (5). Reflecting its established role, the American Urological Association (AUA) guideline lists PPS as a standard oral therapy for IC/BPS, supported by Grade B evidence, indicating that although its therapeutic efficacy may vary, PPS can be effective in selected patients (1). Its mechanism of action involves restoration of the damaged GAG layer, thereby shielding the underlying urothelium from noxious substances in the urine (4, 6). Clinical trials have demonstrated improvements in bladder pain and urinary urgency, thereby establishing PPS as a key component of the therapeutic armamentarium for IC/BPS (7).

Despite its established efficacy, the long-term safety of PPS has become a subject of growing concern. While initial clinical trials reported generally mild adverse effects such as alopecia, diarrhea, and nausea, emerging evidence from post-marketing surveillance and observational studies points towards more severe, previously under-recognized risks. Of particular concern is the association between long-term PPS exposure and a progressive form of pigmentary maculopathy that threatens vision (8, 9).

Pharmacovigilance, the science of detecting, assessing, understanding, and preventing adverse effects, plays a critical role in assessing the safety of medications post-approval (10). Large spontaneous reporting system (SRS) databases, such as the FDA Adverse Event Reporting System (FAERS), are vital for this purpose, providing a real-world evidence to identify potential safety signals that may not have been apparent in premarketing clinical trials (11). Although specific case series and minor studies have examined PPS-associated maculopathy (12), these reports often focus narrowly on ophthalmologic complications, leaving a significant knowledge gap regarding other potential systemic risks. No large-scale pharmacovigilance study has yet analyzed the comprehensive, multi-organ safety profile of PPS using the entirety of the publicly available FAERS database.

Hence, the primary aim of this study is to perform an extensive analysis of the FAERS database spanning more than two decades. This study also characterizes the spectrum of adverse events associated with PPS in the IC/BPS patient population, quantify the reporting risks for these events using well-known pharmacovigilance metrics, and thereby build a robust, data-driven post-marketing safety profile to inform clinical practice, enhance patient counseling, and strengthen regulatory oversight.

## 2 Materials and methods

### 2.1 Data sources

The data for this study were collected from FAERS, which is a public database containing information on adverse event and medication error reports submitted to the FDA. The database is intended to support the agency’s post-marketing safety surveillance program for drugs and therapeutic biologic products. These data can be freely accessed and downloaded from the FDA website.^1^

The database includes demographic and administrative information, initial report image ID numbers, drug information from case reports, reaction data, patient outcomes, and report source information. The downloaded data are organized into multiple tables, including patient demographic information (DEMO), drug information (DRUG), adverse event information (REAC), patient outcome information (OUTC), report source information (RPSR), drug therapy date information (THER), and drug indications (INDI).

The downloaded FAERS tables were processed using SAS software. For example, raw data from the DEMO table were extracted, imported into SAS in DEMO. TXT format, and all the raw data were consolidated into a single table named DEMO. The remaining tables were subsequently imported into SAS sequentially, following the same procedure.

### 2.2 Data deduplication and cleaning

In accordance with the FDA-recommended data deduplication method, the fields PRIMARYID, CASEID, and FDA_DT were selected from the DEMO table. The data were then arranged in ascending order by CASEID, FDA_DT, and PRIMARYID. For reports with the same CASEID, the report with the highest FDA_DT value is retained. Furthermore, for reports where both the CASEID and FDA_DT were identical, the record with the highest PRIMARYID value was kept. Adhering to these deduplication principles, the data cleaning code was executed using SAS.

This procedure was intended to remove duplicate reports—such as those of the same case submitted from different sources—as well as multiple reports for a single case that provide supplementary and updated information. The most recent version of each case report is collected to ensure uniqueness. In addition, only adverse event cases/reports involving suspected drug reactions were included, whereas those related with concomitant or interacting medications were excluded.

The System Organ Class (SOC) and Preferred Term (PT) from the Medical Dictionary for Regulatory Activities (MedDRA, version 27.1) were applied to organize and designate the relevant adverse event (AE) reports. The SOC denotes the category of the AE, while the PT provides standardized terminology for description.

AE reports in which PPS was the primary suspect drug were retrieved by searching for the terms “ELMIRON,” “ELMIRON BLADDER,” “ELMIRON CAP,” “ELMIRON CAPSULE,” “ELMIRON 100,” “ELMIRON 100MG ORAL,” “ELMIRON PENTOSAN POLYSULFATE SODIUM 100 MG,” “ELMIRON PENTOSAN POLYSULFATE SODIUM,” “PENTOSAN POLYSULFATE ELMIRON,” “PENTOSAN POLYSULFATE SODIUM,” “PENTOSAN POLYSULFATE SODIUM CAPSULE” and “PENTOSAN POLYSULFATE SODIUM 100 MG.” To maximize data capture and minimize selection bias arising from missing or incomplete indication fields in FAERS, reports were not filtered based on the indication of use. Therefore, the dataset in this study encompasses all reported exposures, including potential off-label uses. Data were collected for the period from the first quarter of 2004 through the first quarter of 2025. All operations were conducted using SAS software.

### 2.3 Data analysis

#### 2.3.1 Adverse event signal mining for PPS

The primary analytical method used was disproportionality analysis. To confirm the robustness of the findings and decrease false-positive signals, two established algorithms like: the reporting odds ratio (ROR) and the proportional reporting ratio (PRR) were applied. These methods rely on statistical parameters within a 2 × 2 contingency table for signal detection, as shown in Table 1.

**Table 1:** Disproportionality analysis.

2 × 2 table for the signal detection				2 × 2 table for the signal detection of gender differences			
Database	Target AE	All other AE	Total	Database	Reports with target AE	Reports with All other AE	Total
Target drug	a	b	a + b	Female	a^†^	b^†^	a^†^ + b^†^
All other drugs	c	d	c + d	Male	c^†^	d^†^	c^†^ + d^†^
Total	a + c	b + d	a + b + c + d	Total	a^†^ + c^†^	b^†^ + d^†^	a^†^ + b^†^ + c^†^ + d^†^

Formulas and threshold values of ROR and PRR		
Method	Formulas	Inclusion criteria for AE
ROR	$\mathrm{ROR}=\frac{\left(a/c\right)}{\left(b/d\right)}$	Lower limit of95%CI>1 a≥3
	$\mathrm{SE}(\ln R0R)=\sqrt{\frac{1}{a}+\frac{1}{b}+\frac{1}{c}+\frac{1}{d}}$	
	$95\%\mathrm{CI}=e^{\ln(R0R)\pm 1.96\sqrt{\frac{1}{a}+\frac{1}{b}+\frac{1}{c}+\frac{1}{d}}}$	
PRR	$\mathrm{PRR}=\frac{a/(a+b)}{c/(c+d)}$	PRR≥2 χ2≥4 a≥3
	$\mathrm{SE}(\ln\mathrm{PRR})=\sqrt{\frac{1}{a}-\frac{1}{a+b}+\frac{1}{c}-\frac{1}{c+d}}$	
	$95\%\mathrm{CI}=e^{\ln(\mathrm{PRR})\pm 1.96\sqrt{\frac{1}{a}-\frac{1}{a+b}+\frac{1}{c}-\frac{1}{c+d}}}$	
	$\chi 2=\frac{{\left(\mathrm{ad}-\mathrm{bc}\right)}^{2}(a+b+c+d)}{\left(\;a+b)(a+c)(c+d)(b+d\right)}$	

a, the number of reports containing both the target drug and target AE; b, the number of reports containing other AE of the target drug; c, the number of reports containing the target AE of other drugs; d, the number of reports containing other drugs and other AE. a^†^ is the number of reports of the target adverse event in females; b^†^ is the number of reports of other adverse events in females; c^†^ is the number of reports of the target adverse event in males; and d^†^ is the number of reports of other adverse events in males.

The ROR and PRR values show the strength of the signal; higher value signifies a stronger association between the target drug and the target AE. Further, a signal was considered positive if the following criteria were met:

(1) The number of reports (a) ≥ 3;
(2) For ROR, the lower limit of the 95% confidence interval (CI) > 1; and.
(3) For PRR, the value ≥2 and χ^2^ ≥ 4.

#### 2.3.2 Classification of patient outcomes

Patient outcomes were characterized according to the FDA standards. Serious adverse events (SAEs) were defined as those resulting in one or more of the following outcomes: death, life-threatening condition, hospitalization (initial or prolonged), disability or permanent damage, congenital anomaly/birth defect, or other serious (medically important events). The “Other Serious” category includes events that may not result in death, hospitalization, or disability but could jeopardize the patient or may require medical or surgical intervention to prevent such outcomes. All remaining outcomes were classified as non-serious.

#### 2.3.3 Adverse event signal mining for gender differences

Disproportionality analysis was also maintained as the primary analytical method, but with a different 2 × 2 table for the signal detection of gender differences, as shown on the right side of Table 1. The variables are defined as follows: a^†^ is the number of reports of the target adverse event in females; b^†^ is the number of reports of other adverse events in females; c^†^ is the number of reports of the target adverse event in males; and d^†^ is the number of reports of other adverse events in males.

Based on this 2 × 2 table, the ROR and its 95% confidence interval (CI) were calculated using the following formula: ROR = (a^†^/c^†^)/(b^†^/d^†^). The criteria for signal detection were as follows:

(1) Number of reports (a) ≥ 3.
(2) ROR > 1 and lower limit of the 95% CI > 1, indicating that females are more likely to report a specific adverse event compared to males. The larger the ROR value, the stronger the association. If ROR < 1 and upper limit of the 95% CI < 1, it suggests that males are more likely to report the adverse event. The smaller the ROR value, the stronger the association.

#### 2.3.4 Time to onset (TTO) analysis

In this study, TTO of ADEs associated with PPS was defined as the time interval between the ADE occurrence date (EVENT_DT) in the DEMO file and the drug start date (START_DT) in the THER file. Cases with inaccurate or missing dates, as well as those where the ADE date preceded the drug start date, were excluded.

To comprehensively characterize the temporal distribution of these ADEs, a series of survival analyses were conducted. First, the TTO for all ADEs were analyzed collectively, and the cumulative probability of events was visualized using the non-parametric Kaplan -Meier method.

Second, to quantitatively describe the pattern of hazard over time, three candidate parametric models were fitted to the TTO data: the Weibull, log-normal, and log-logistic models. The best-fitting model was selected based on the Akaike Information Criterion (AIC) and Bayesian Information Criterion (BIC). Lower AIC and BIC values indicate a better fit of the model to the data.

**Figure 1:**
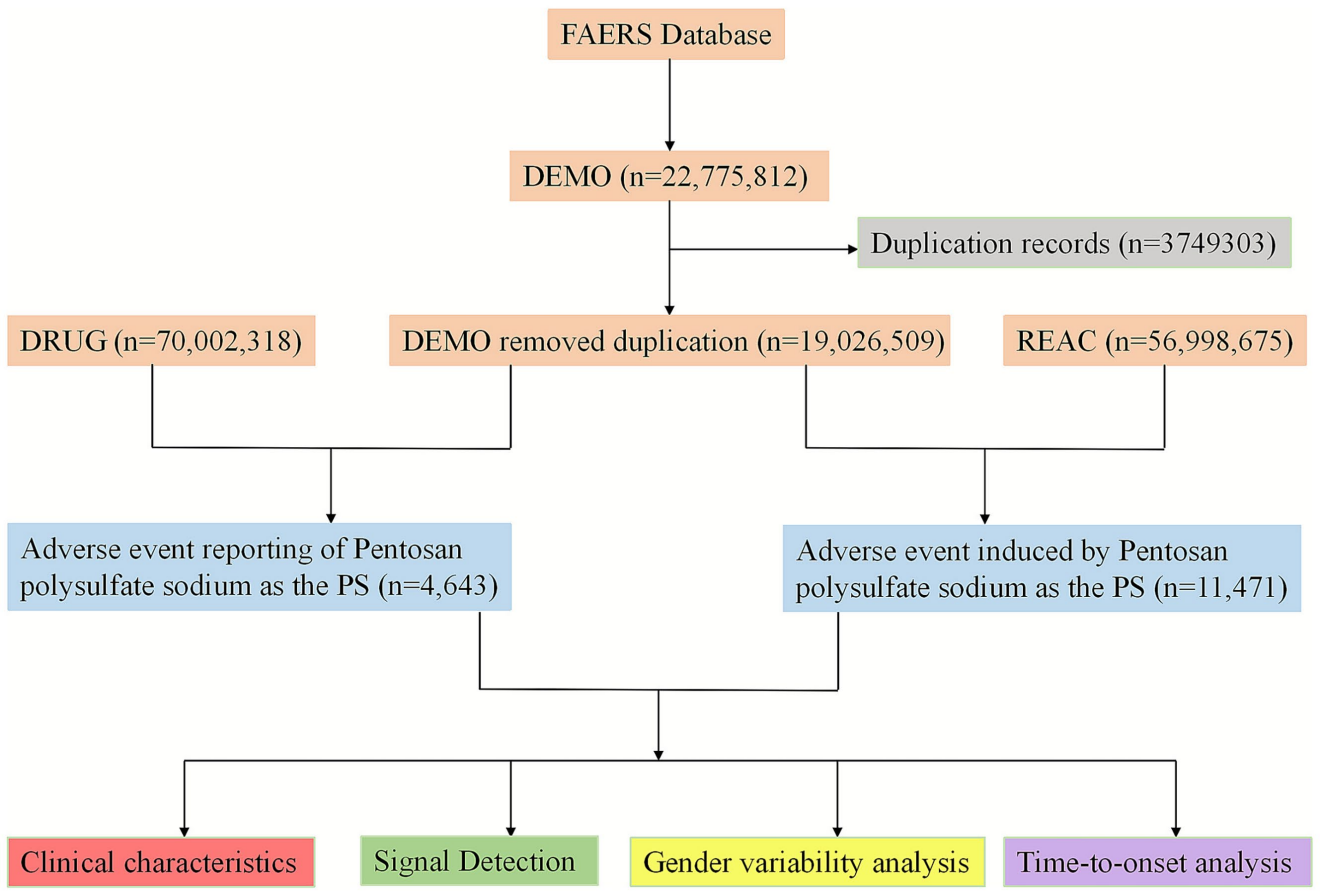
Flowchart of the study. DEMO, demographic and administrative information; DRUG, drug information; REAC, preferred terminology for adverse event; PS, primary suspect drug.

Among these models, the Weibull distribution is particularly important for understanding hazard trends, which are defined by a scale parameter (*α*) and a shape parameter (*β*). For the purposes of this study, only the parameter *β* was considered and discussed, as it directly reflects the change in the hazard rate: if the shape parameter *β* < 1 and its 95% confidence interval (CI) upper limit is less than 1, the hazard decreases over time (early failure pattern); if *β* ≈ 1 and its 95% CI includes 1, the hazard is constant (random failure pattern); and if *β* > 1 and its 95% CI lower limit is greater than 1, the hazard increases over time (wear-out failure pattern). The flowchart of this study is shown in Figure 1.

## 3 Results

### 3.1 Adverse event reporting for PPS

After cleaning the FAERS data from the first quarter of 2004 to the first quarter of 2025, a total of 11,471 reports were identified in which PPS was the primary suspect drug. Among the reported adverse events, the male-to-female ratio was 1:12.32, and the age distribution was predominantly concentrated in the 18–64 age group (27.8%). The primary reporters were lawyers (49.2%) and consumers (30.1%), with the reports mainly submitted from the United States (97.3%) and Canada (1.4%). The annual number of adverse event reports generally trended upward over the study period, peaking in 2022 and 2023, at 1,026 cases each year. The baseline characteristics of the AE reports are summarized in Table 2.

**Table 2:** Clinical characteristics of PPS-related events reported in FAERS.

Characteristics		Case number (*n*)	Case proportion (%)	Characteristics	Case number (*n*)	Case proportion (%)
				Year		
Gender	Male	329	7.1	2025	72	1.6
	Female	4,052	87.3	2024	643	13.8
	Unknown	262	5.6	2023	1,026	22.1
Age	<18	616	13.3	2022	1,026	22.1
	18–64	1,289	27.8	2021	325	7.0
	≥65	476	10.2	2020	221	4.8
	Unknown	2,262	48.7	2019	169	3.6
Reported person	Physician	206	4.4	2018	164	3.5
	Consumer	1,398	30.1	2017	125	2.7
	Health professional	518	11.2	2016	133	2.9
	Other health-professional	98	2.1	2015	372	8.0
	Pharmacist	112	2.4	2014	40	0.9
	Lawyer	2,286	49.2	2013	40	0.9
	Unknown	25	0.5	2012	59	1.3
Reported countries (top two)	America	4,514	97.3	2011	71	1.5
	Canada	68	1.4	2010	35	0.8
Outcome	Death	15	0.3	2009	20	0.4
	Disability	43	0.9	2008	18	0.4
	Hospitalization	97	2.1	2007	18	0.4
	Life-threatening	10	0.2	2006	26	0.6
	Other serious outcome	3,160	68.1	2005	22	0.5
	Unknown	1,318	28.4	2004	18	0.4

### 3.2 Top 20 adverse events with signals for PPS ranked by number of reports

**Figure 2:**
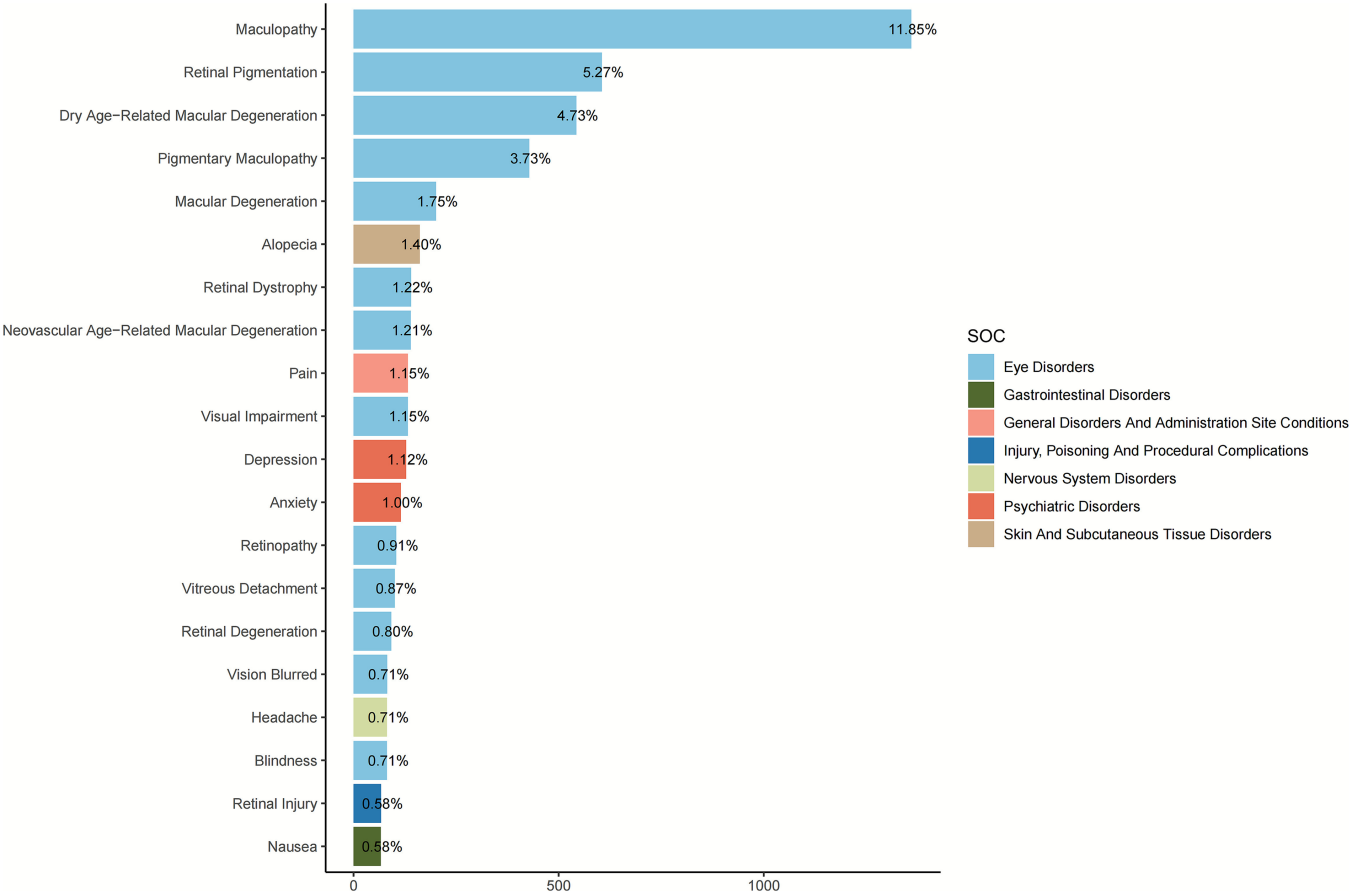
Top 20 reported preferred terms (PTs) associated with PPS (ranked by number of reports).

A disproportionality analysis was conducted to detect signals for adverse events (AEs) where PPS was the primary suspect drug. The analysis revealed that adverse events within the eye disorders SOC were overwhelmingly the most prominent signals (Figure 2). Specifically, maculopathy was the single most reported AE, accounting for 11.85% of all cases. A constellation of other PTs related to retinal pathology also demonstrated high reporting frequencies, including: retinal pigmentation (5.27%), dry age-related macular degeneration (4.73%), pigmentary maculopathy (3.73%), macular degeneration (1.75%), retinal dystrophy (1.22%), and neovascular age-related macular degeneration (1.21%). Furthermore, other significant vision-related AEs such as visual impairment (1.15%), retinopathy (0.91%), vitreous detachment (0.87%), retinal degeneration (0.80%), vision blurred (0.71%), and blindness (0.71%) were also identified within the top reported events.

In addition to ocular events, signals were detected in other SOCs. The most frequent non-ocular AE was alopecia, categorized under skin and subcutaneous tissue disorders, with a reporting percentage of 1.40%. Notable signals from psychiatric disorders included depression and anxiety, with reporting frequencies of 1.12 and 1.00%, respectively. Other reported AEs included pain (1.15%), headache (0.71%), retinal injury (0.58%), and nausea (0.58%).

### 3.3 Analysis of the top 3 adverse events ranked by signal strength (ROR) for PPS under the SOCs

**Figure 3:**
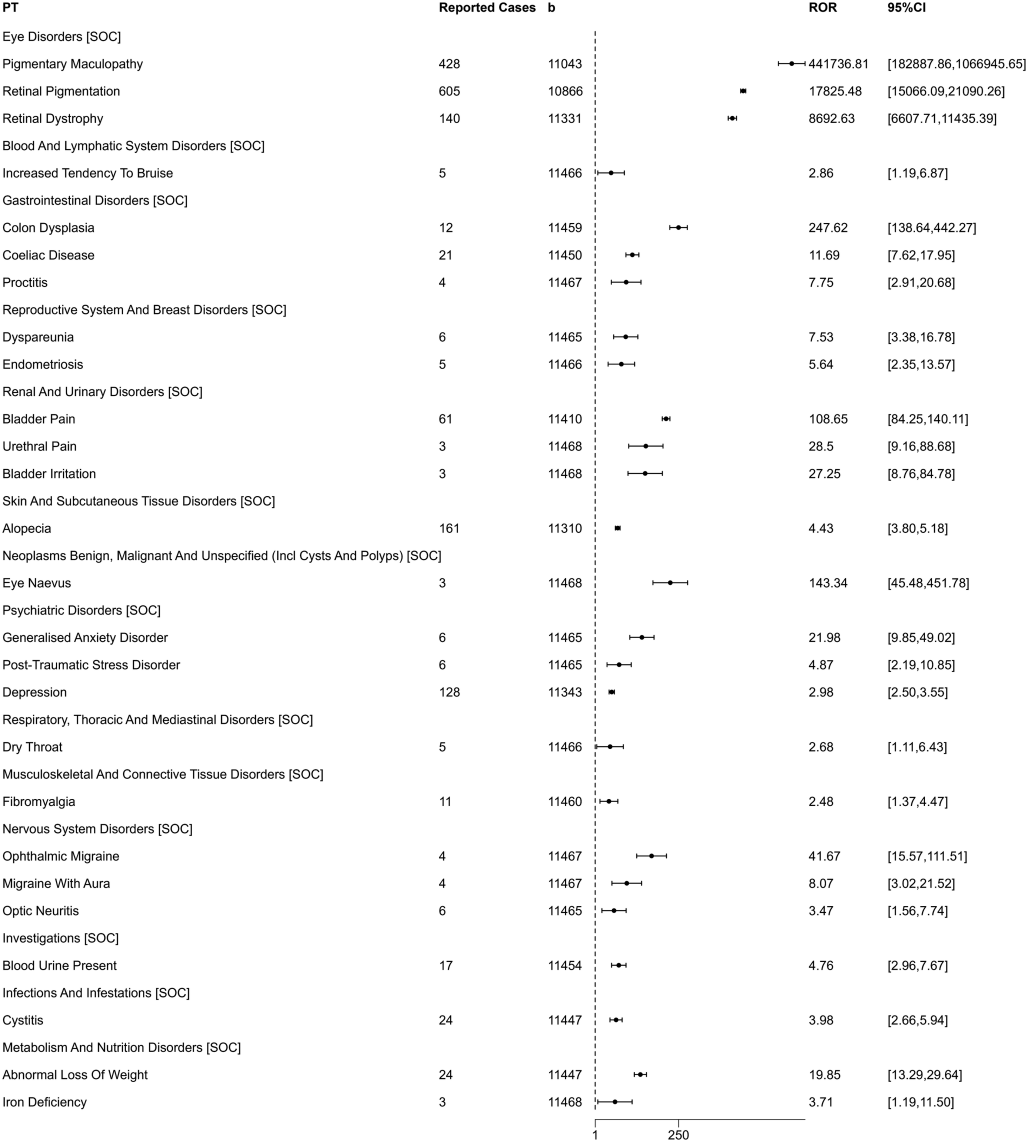
Analysis of the top 3 adverse events reported for PPS under the SOCs (ranked by ROR signal strength).

AE reports in which PPS was identified as the primary suspect drug were analyzed, mapping the PTs to their corresponding SOCs. The analysis revealed that the most significant and strongest signals were overwhelmingly concentrated in the eye disorders SOC (Figure 3). Pigmentary maculopathy demonstrated an exceptionally strong signal with an ROR of 441,736.81 (95% CI: 182,887.86–1,069,945.65, PRR: 425,254.99), based on 428 reported cases. Similarly, retinal pigmentation showed a very high disproportionality (ROR = 17,825.48; 95% CI: 15,066.69–21,090.26, PRR: 16,885.39) with 605 cases, and retinal dystrophy also presented a significant risk signal (ROR = 8,892.63; 95% CI: 6,607.71–11,435.39, PRR: 8,586.55) from 140 cases.

In addition to these powerful ocular signals, the analysis uncovered significant risks across a range of other organ systems. A particularly strong signal was identified within gastrointestinal disorders for colon dysplasia, with an ROR of 247.62 (95% CI: 138.64–442.27, PRR: 247.37). Of direct clinical relevance, several AEs within the renal and urinary disorders SOC exhibited significant signals, including bladder pain (ROR = 108.65; 95% CI: 84.25–140.11, PRR: 108.08), urethral pain (ROR = 28.5; 95% CI: 9.16–88.68, PRR: 28.5), and bladder irritation (ROR = 27.25; 95% CI: 8.76–84.78, PRR: 27.25). Furthermore, the previously noted AE of alopecia was confirmed as a significant signal (ROR = 4.43; 95% CI: 3.80–5.18, PRR: 4.39) with 161 cases. Other notable signals included ophthalmic migraine in nervous system disorders (ROR = 41.67; 95% CI: 15.57–111.51, PRR: 41.66), generalized anxiety disorder in psychiatric disorders (ROR = 21.98; 95% CI: 9.85–49.02, PRR: 21.97), and abnormal loss of weight in metabolism and nutrition disorders (ROR = 19.85; 95% CI: 13.29–29.64, PRR: 19.81).

Given that PPS is indicated for patients aged 16 years and older, a sensitivity analysis was executed to assess the robustness of the detected signals. The data were reanalyzed by including only reports with a documented age of ≥16 years, excluding pediatric cases and reports with missing age information. The analysis confirmed that the signals for key ocular adverse events remained exceptionally strong and significant in the adult-only cohort. Specifically, pigmentary maculopathy showed an ROR of 237,189.57 (95% CI: 97,469.39–577,195.45, PRR: 229,361.85); retinal pigmentation presented an ROR of 16,425.40 (95% CI: 13,002.78–20,748.92, PRR: 15,677.02); and retinal dystrophy demonstrated an ROR of 9,062.42 (95% CI: 6,310.95–13,013.48, PRR: 8,943.48). These results corroborate the primary findings and specify that the identified risks are not driven by off-label pediatric use or data with unknown demographics.

### 3.4 Patient outcomes

An analysis was conducted on the patient outcomes from AE reports where PPS was the primary suspect drug. The clinical outcomes for the reported PPS-related adverse events are shown in Table 1.

Analysis of the patient outcomes revealed that a majority of the cases were classified as serious. The most frequently reported category was ‘Other Serious Outcome’ accounting for 68.1% (*n* = 3,160) of all reports. A significant portion of cases, 28.4% (*n* = 1,318), had an unknown outcome.

Among the specifically defined severe outcomes, hospitalization was reported in 2.1% of cases (*n* = 97). Disability was the outcome in 0.9% of the reports (*n* = 43). The most severe outcomes, death and life-threatening events, were reported less frequently, accounting for 0.3% (*n* = 15) and 0.2% (*n* = 10) of cases, respectively.

### 3.5 Gender differences analysis (visualization of signal results)

**Figure 4:**
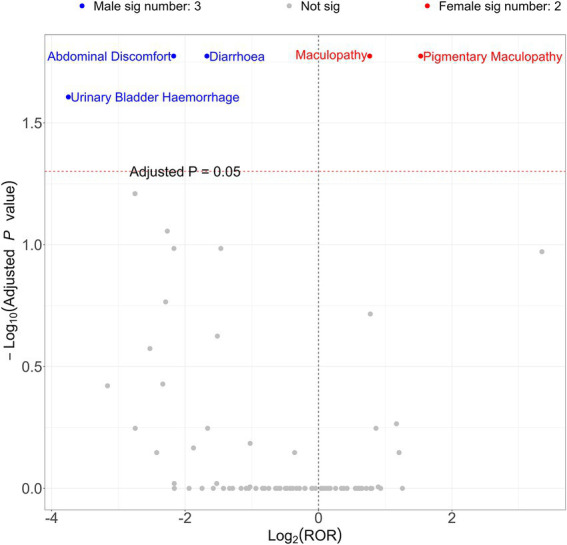
Volcano map of gender differences analysis of PPS.

To analyze the gender-specific ADE signal results for PPS, a volcano plot for intuitive visualization was generated (Figure 4). The *y*-axis of the volcano plot represents the −log10 (adjusted *p*-value), while the *x*-axis represents the Log_2_(ROR) value. Further, each point in the plot represents a PPS-related ADE. The ADEs that are statistically significant are labelled, defined as signals with a *p*-value <0.05 after adjustment using the false discovery rate (FDR) method. Three significant signals were observed in males, including abdominal discomfort, diarrhea, and urinary bladder hemorrhage. In females, two strong signals were observed: maculopathy and pigmentary maculopathy.

### 3.6 TTO analysis of overall ADEs and at the PT level for eye disorders

**Figure 5:**
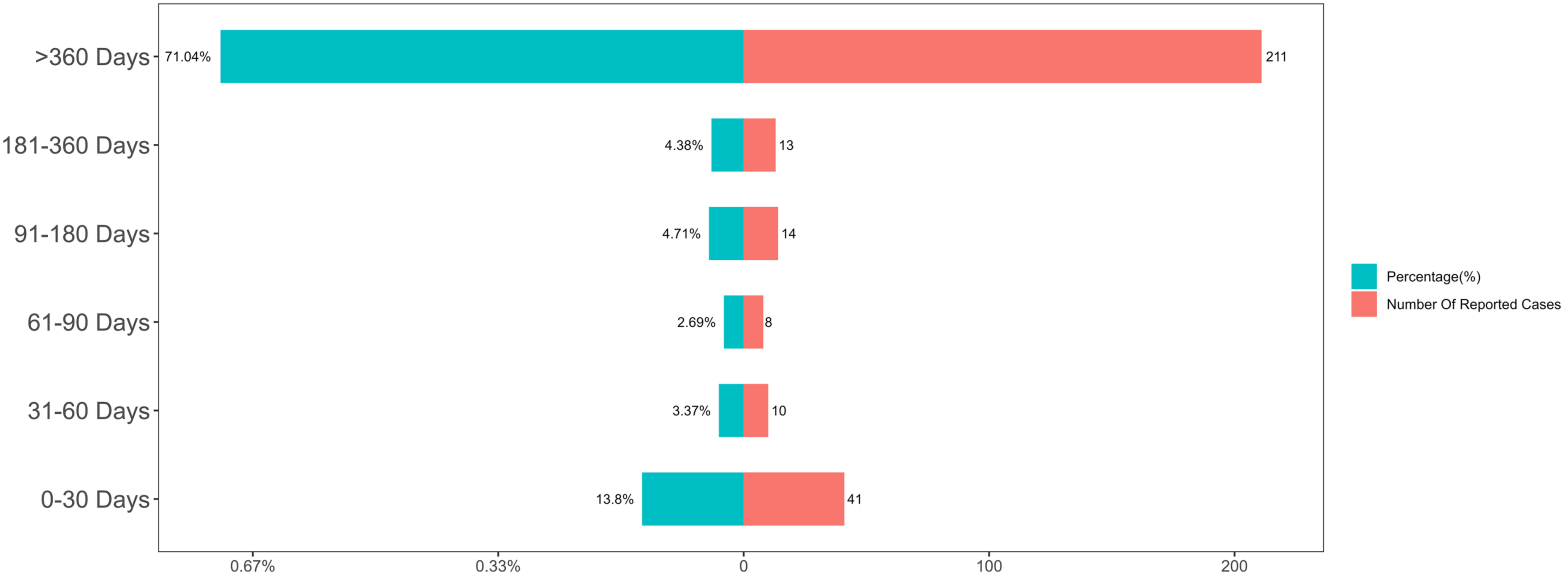
Distribution of time-to-onset (TTO) for overall PPS-related adverse events.

**Figure 6:**
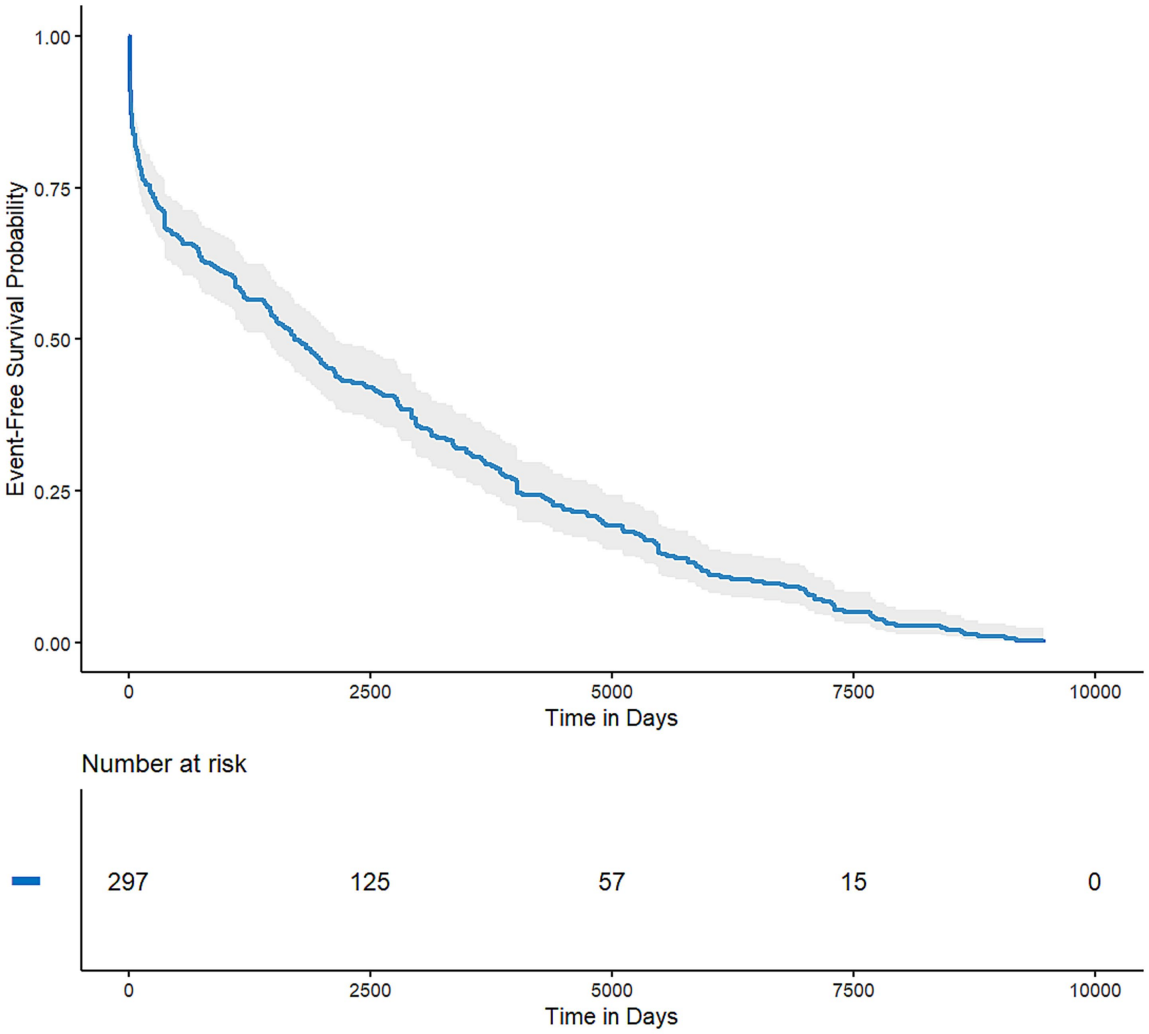
Kaplan–Meier curve for the cumulative probability of remaining free of adverse events (the table titled “Number at Risk” below the graph displays the number of patients who have not yet reported an adverse event or been censored at specific time intervals).

After excluding reports with inaccurate, missing, or unknown onset dates, a total of 297 cases were included in the TTO analysis. For the overall cohort of PPS-related ADEs, the median TTO was 1,715 days, with a wide interquartile range (IQR) of 212 to 4,018 days. As shown in Figure 5, while an initial portion of cases occurred within the first month of PPS use (*n* = 41, 13.8%), the majority of events (71.04%) were reported after 1 year of treatment. It is worth noting that even after the first year of PPS therapy, adverse drug events were still likely to occur. This long-term risk profile is clearly illustrated by the non-parametric Kaplan–Meier survival curve, which shows a gradual and continuous decline in event-free probability over an extended period (Figure 6). As shown in the risk table titled ‘Number at Risk’ beneath the Kaplan–Meier curve, these numbers represent the count of patients who had not yet experienced an adverse event and remained under observation at each specific time point.

To quantitatively describe the hazard pattern over time, we compared three parametric models. The Weibull distribution was selected as the optimal model, as it demonstrated a superior fit compared to the log-normal and log-logistic distributions, evidenced by its substantially lower Akaike Information Criterion (AIC) of 5,138.7 (Table 3). The shape parameter (*β*) of the Weibull model was 0.62 (95% CI: 0.56–0.69). As the *β* value and its 95% CI upper limit were both less than 1, this indicates a decreasing hazard rate over time, suggesting that the risk of an ADE, while prolonged, is highest during the earlier stages of long-term therapy and gradually subsides.

**Table 3:** Comparison of goodness-of-fit for candidate parametric models.

Model	AIC	BIC	Anderson–Darling statistics
Weibull distribution	5138.7	5146.1	9.06
Log-logistic distribution	5239.4	5246.8	13.12
Log-normal distribution	5248.9	5256.3	16.28

**Figure 7:**
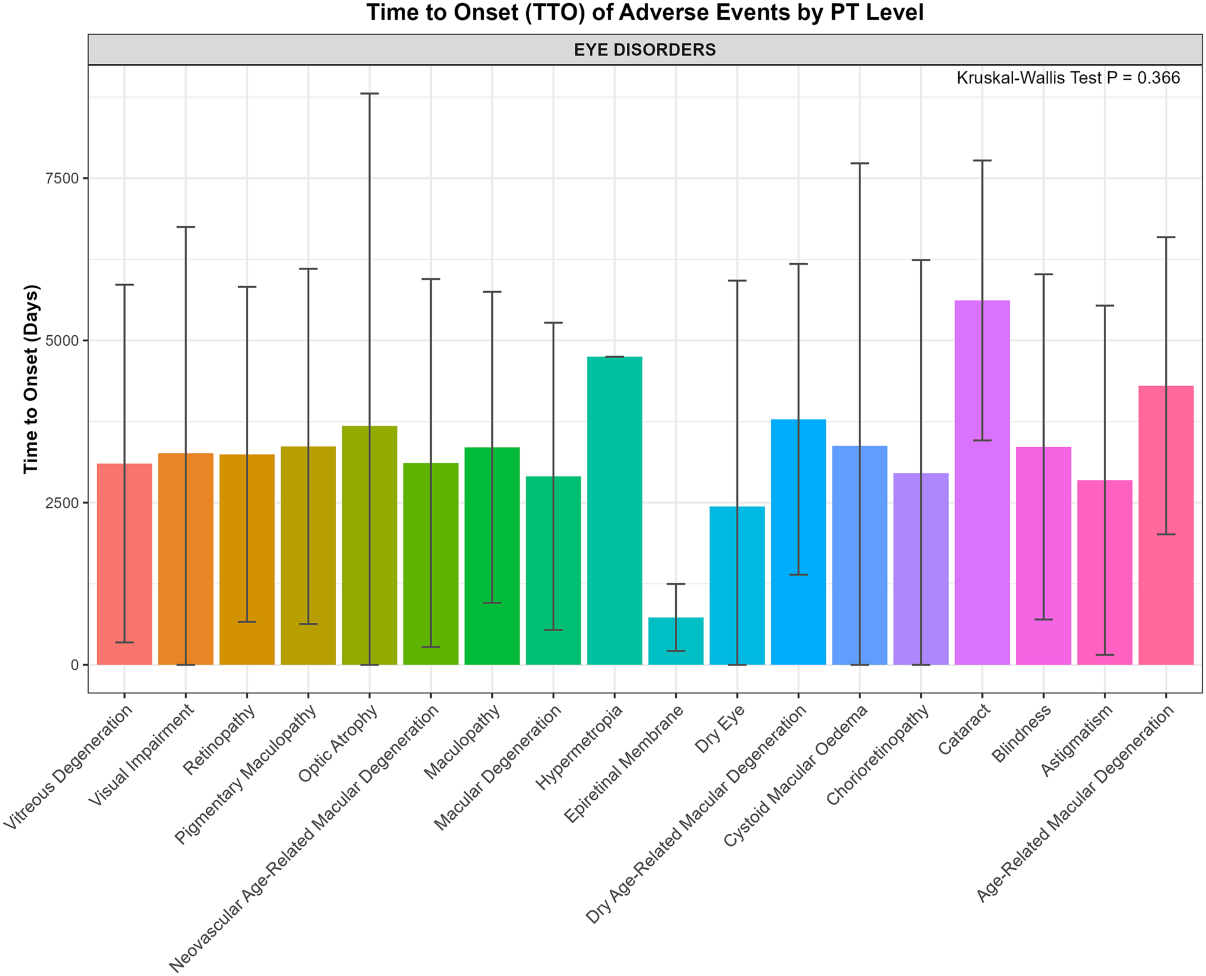
Mean time-to-onset (TTO) for specific preferred terms (PTs) within the ‘Eye disorders’ SOC.

Given the clinical significance of ophthalmologic complications, we further investigated the TTO for various PTs within the ‘Eye disorders’ SOC to determine if their onset times differed. However, the Kruskal-Wallis test showed no statistically significant difference in the TTO distributions among these 18 PTs (*p* = 0.366). Although there was no significant difference among the PTs, it is noteworthy that the mean TTO for the majority of these ophthalmologic events exceeded 2,500 days (approximately 6.8 years) (Figure 7).

## 4 Discussion

This study signifies a large-scale pharmacovigilance investigation over a long time span for adverse reactions associated with PPS since its market release, based on the FAERS database. The primary aim of this research is to provide a detailed analysis of the AEs related to PPS reported to date. The results presented in this study offer valuable and precise insights into the safety profile of PPS in a real-world clinical setting.

Based on the clinical characteristics, a markedly higher frequency of adverse reactions to PPS was observed in females (87.3%) compared to males (7.1%). This is attributed to the higher susceptibility of females to interstitial cystitis/bladder pain syndrome (IC/BPS) (13), which consequently increases their likelihood of receiving treatment with the drug. AEs associated with PPS were primarily concentrated among patients aged 18–64 years and the elderly; however, with 2,262 cases (48.7%) lacking patient age information, it is difficult to determine if the risk is higher in the elderly.

The number of AE reports from the United States significantly exceeds that of other countries. While the FAERS database theoretically includes global data, it is predominantly supplied by reports from the U.S. (14). This disproportionate reporting can be attributed to several factors, including a larger medication-using population, a stronger reporting culture, earlier market entry, and broader indications. Perhaps most critically, PPS is the only oral medication approved by the FDA specifically for the treatment of IC/BPS, and it is recommended as a standard oral therapy with Grade B evidence by the American Urological Association. These factors have collectively promoted the widespread use of the drug in the United States.

Analyzing the reporting timeline discloses a dramatic spike in adverse event reports in 2022 and 2023 (1,026 cases each year), representing a more than three-fold increase compared to 2021. This surge is unlikely to stem from a sudden expansion of the patient population or marketing in new regions, as 97.3% of our data consistently originated from the United States. Instead, this pattern is characteristic of ‘notoriety bias’ (or stimulated reporting). Following the FDA’s labeling update in 2020 to include warnings about pigmentary maculopathy, there was a significant increase in public awareness and product liability litigation in the U.S. The fact that lawyers constituted the largest group of reporters (49.2%) in this study strongly supports the conclusion that the 2022 peak was driven by retrospective reporting facilitated by legal professionals rather than a prospective increase in clinical incidence.

Regarding established safety risks, disproportionality analysis corroborated the well-documented association of ophthalmologic complications, with maculopathy and other retinal pathologies dominating the top-ranked signals. This confirmation of known risks reinforces the validity of our dataset and analytical approach. Similarly, alopecia–a recognized side effect of PPS–was identified as the most frequently reported non-ocular AE. This finding provides strong, real-world evidence that quantifies its high reporting frequency in a large patient population, reinforcing its clinical significance alongside the ocular AEs.

In addition to confirming known risks, a key contribution of this study is the identification of potential new safety signals not currently listed in the drug’s label. The most notable among these were depression and anxiety within psychiatric disorders. A review of the most recent FDA-approved Medication Guide for PPS, dated March 12, 2021, reveals that while these specific psychiatric risks are not included,^2^ they emerged as significant signals in our analysis. This discrepancy underscores the indispensable role of long-term, post-marketing pharmacovigilance. Real-world data, gathered over extended periods and from diverse patient populations, can uncover potential safety signals that were not apparent during pre-market clinical trials. Therefore, these findings suggest that the safety profile of PPS may be broader than currently documented, and the prescribing information could warrant further review and potential updates to include these psychiatric signals.

Disproportionality analysis at the SOC level found that the most striking signals were overwhelmingly concentrated in the ‘Eye Disorders’ SOC. The exceptionally high ROR for pigmentary maculopathy (ROR = 441,736), along with similarly strong signals for retinal pigmentation and retinal dystrophy, provides unequivocal real-world evidence supporting the well-documented link reported in clinical studies between long-term PPS use and a specific, vision-threatening maculopathy. The magnitude of these ROR values suggests a very strong and specific drug-event association, positioning maculopathy reported with PPS as the most critical safety concern for patients undergoing this therapy.

A major limitation that must be carefully considered when interpreting our findings is the profound potential for reporting bias. Nearly half of the reports (49.2%) were submitted by lawyers. This suggests a strong litigation bias, also known as notoriety bias, where reporting is heavily skewed towards well-publicized and litigated adverse events. Consequently, the exceptionally high ROR observed for pigmentary maculopathy (ROR > 400,000) is likely inflated and may not reflect the true reporting ratio in the general patient population, but rather the focused reporting of this specific compensable harm. This bias must temper the interpretation of the magnitude of all signals, especially the ocular findings.

While the ocular toxicities are a known risk, this study also brings to light other significant signals across different organ systems that warrant clinical attention. The detection of a strong signal for colon dysplasia within gastrointestinal disorders is a novel and potentially serious finding that requires further investigation to understand any potential pathological link. Additionally, the significant signals for bladder pain, urethral pain, and bladder irritation are noteworthy. While these events could be manifestations of the underlying condition (interstitial cystitis/bladder pain syndrome) and thus subject to protopathic bias, their strong disproportionality suggests that they could also represent a paradoxical worsening of symptoms or distinct AEs related to PPS therapy, a possibility that should not be dismissed.

Furthermore, the identification of strong signals for ophthalmic migraine, generalized anxiety disorder, and abnormal loss of weight highlights the systemic impact of PPS beyond its intended site of action. These findings broaden the standard safety profile of the drug and emphasize the need for clinicians to be aware of a wider range of potential AEs when monitoring patients on long-term PPS treatment.

The analysis of patient outcomes underscores the clinical severity of AEs associated with PPS, as the majority of reports were classified as serious. The predominance of the ‘Other Serious Outcome’ category (68.1%) warrants particular attention. This FDA classification typically encompasses events that result in substantial disruption of life activities, persistent or significant disability or incapacity, or require medical intervention to prevent a more severe outcome. For a drug like PPS, where primary ADEs such as pigmentary maculopathy are chronic and progressive, this category likely captures the significant, long-term functional impairment–most notably vision impairment–that profoundly impacts patients’ quality of life.

While less frequent, the occurrence of hospitalization (2.1%), disability (0.9%), and even death (0.3%) confirms the potential for substantial morbidity. Collectively, this data indicates that the primary clinical burden of PPS-related ADEs is chronic, disabling morbidity rather than acute mortality, reinforcing the need for proactive monitoring to mitigate these serious, life-altering outcomes.

A comparative analysis of gender-specific ADE signals for PPS was also conducted. This stratified analysis showed distinct risk profiles between males and females. In females, the most significant signals were for maculopathy and pigmentary maculopathy, which is consistent with the overall findings of this study. This observation is likely influenced by the fact that interstitial cystitis, the primary indication for PPS, is overwhelmingly more prevalent in women (15). The resulting higher number of female patients exposed to the drug naturally contributes to a greater number of reported ocular events and, consequently, stronger statistical signals in this group.

Interestingly, a different set of signals emerged for male patients, namely abdominal discomfort, diarrhea, and urinary bladder hemorrhage. The emergence of these distinct gastrointestinal and bleeding-related signals in males suggests that sex-based biological factors may modulate the adverse effects of PPS. The mechanisms underlying sex differences in ADEs are multifactorial and can be attributed to differences in both pharmacokinetics (what the body does to the drug) and pharmacodynamics (what the drug does to the body) (16).

It is well-established that men and women differ in body composition, such as body weight and percentage of body fat, and physiological functions, including gastric emptying time and renal clearance, all of which can alter a drug’s absorption, distribution, and elimination (17). For instance, differences in gastrointestinal transit time or gastric pH could potentially influence the local effects of orally administered PPS, possibly contributing to the gastrointestinal signals observed in males (18, 19). Furthermore, pharmacodynamic differences, often influenced by sex hormones like estrogen and testosterone, can affect drug-metabolizing enzymes (e.g., Cytochrome P450 enzymes) and target receptor sensitivity (20). It is plausible, though speculative, that hormonal differences could influence pathways related to coagulation or mucosal integrity, potentially predisposing males to the specific hemorrhagic and gastrointestinal ADEs identified in our analysis.

This gender-specific analysis adds a crucial, nuanced perspective to the safety profile of PPS. While the predominant risk of ocular toxicity appears most prominent in the largely female patient population, distinct signals in males suggest that risk profiles are not uniform across sexes. These findings highlight the importance of considering sex as a biological variable in pharmacovigilance and suggest that future mechanistic studies are warranted to elucidate why these gender-specific adverse reactions occur.

A central finding is the characterization of the exceptionally long latency period for PPS-associated AEs. The median TTO of 1,715 days (approximately 4.7 years) for the overall cohort, combined with the observation that over 70% of events occur after the first year of therapy, provides strong, quantitative evidence for a long-term, cumulative risk profile. This profile is illustrated by the non-parametric Kaplan–Meier survival curve, which displays an initial steeper decline in event-free probability followed by a more gradual and prolonged decrease over the subsequent years. This shape is the classic visual representation of a decreasing hazard rate.

This visual evidence is quantitatively confirmed by the parametric modeling. The Weibull distribution, identified as the optimal model, yielded a shape parameter (*β*) of 0.62, consistent with a decreasing hazard rate over time. This ‘early failure’ pattern is likely driven by common, early-onset adverse reactions such as gastrointestinal disturbances and alopecia. However, this statistical trend for the aggregate dataset does not contradict the clinical reality of PPS-associated maculopathy. Unlike the early-onset events that influence the overall *β* value, the ocular toxicity shows a distinct, long-latency profile (cumulative toxicity) with a median onset of several years, as evidenced by the Kaplan–Meier curve’s extended tail and the specific TTO analysis for eye disorders. Consequently, while the overall probability of experiencing a new AE is mathematically highest in the earlier phases, the specific risk for vision-threatening pathologies persists and likely increases with cumulative exposure. This complex pattern underscores the need for both early vigilance and sustained, long-term patient monitoring.

Analysis of specific ophthalmologic ADEs reinforces this overarching conclusion. The Kruskal–Wallis test showed no statistically significant difference in the onset times among the 18 different types of eye disorders (*p* = 0.366). This non-significant result is clinically meaningful, indicating that no single, early-onset ocular event can serve as a sentinel warning for later, more severe pathologies. Instead, the critical finding is the shared characteristic of these events: the mean TTO for the majority of these serious ocular complications exceeded 2,500 days (nearly 7 years). This reinforces the concept that ocular toxicity observed with PPS is a phenomenon of long-term cumulative exposure, underscoring the absolute necessity for vigilant, long-term ophthalmologic screening in all patients receiving this therapy, regardless of the specific type of ocular pathology that may eventually develop.

These findings are broadly consistent with the growing body of literature that has firmly established the link between long-term PPS use and a unique, vision-threatening maculopathy (21–23). The exceptionally strong signals for maculopathy-related PTs in our study corroborate the clinical observations from numerous case series and cohort studies. While clinical studies have estimated the prevalence of this condition to be as high as 20% in long-term users (24), this pharmacovigilance study complements those findings by demonstrating the sheer strength of the drug-event association on a population level. Furthermore, the characterization of a mean onset time exceeding 6.8 years for most ophthalmologic ADEs provides large-scale, real-world data supporting the concept of a dose- and duration-dependent toxicity, a theme recurrent throughout the clinical literature (25). A particularly concerning finding, echoed in recent studies, is that the maculopathy may continue to progress even after drug cessation (26–28).

The clinical implications of these findings, contextualized by current research, are direct and compelling. There is a clear and urgent need to establish universal, evidence-based screening guidelines for long-term PPS therapy patients. TTO analysis indicates that screening should begin within the first few years of treatment and continue vigilantly thereafter. Modalities such as optical coherence tomography (OCT) and fundus autofluorescence (FAF), as described in recent literature, are crucial for detecting early structural changes, and newer techniques like OCT angiography may enable even earlier detection of choriocapillaris flow deficits before significant RPE damage is apparent [29–31]. The irreversible and progressive nature of this damage elevates this primary clinical goal from treatment to prevention and early detection through proactive monitoring.

Several avenues for future research emerge from these findings. First, well-designed epidemiological studies are needed to confirm the causal relationship between PPS and the novel signals for depression and anxiety. Second, further investigation is required to determine cumulative dose and duration thresholds that confer the highest risk for maculopathy, thereby refining screening protocols and informing patient–physician discussions. Finally, basic science research is essential to elucidate the underlying pathophysiological mechanisms of PPS-associated retinal toxicity, which remain poorly understood but are hypothesized to involve direct RPE toxicity or disruption of the choriocapillaris (32, 33). Addressing these questions will be critical to developing effective risk mitigation strategies for patients who rely on PPS for the interstitial cystitis management.

Beyond the identified novel signals, the risk of hemorrhage warrants attention, given the pharmacological profile of PPS as a weak heparin analogue. Although the primary analysis highlighted ocular and psychiatric signals, a specific signal for urinary bladder hemorrhage was detected in the male cohort (Figure 4). The risk of bleeding complications may be exacerbated by the concomitant use of anticoagulants, thromboprophylaxis drugs, or nonsteroidal anti-inflammatory drugs (NSAIDs). Clinicians must exercise caution when prescribing PPS to patients receiving these therapies. To ensure patient safety, doctors should reassess the patient’s progress and potential bleeding risks every 3 months. This regular monitoring is crucial for determining the appropriate duration of Elmiron treatment and for promptly managing any emerging AEs.

This study has several limitations inherent to pharmacovigilance analyses of spontaneous reporting systems like FAERS. First, the data is subject to significant reporting biases. The vast majority of reports originated from the United States (97.3%) and were predominantly submitted by lawyers (49.2%) and consumers (30.1%), suggesting a strong influence of litigation and public awareness rather than purely clinical observation. Consequently, caution should be exercised when generalizing these conclusions to other geographical regions or broader patient populations. Second, reports often contain incomplete or inaccurate information regarding crucial variables like cumulative dosage, precise treatment duration, and comorbidities, limiting detailed risk-factor analysis. Third, the absence of a denominator representing the total number of exposed patients means that true incidence rates cannot be calculated; the findings, including RORs, represent statistical associations and do not establish causality. They should be interpreted as hypothesis generating.

## 5 Conclusion

In conclusion, this comprehensive 21-year real-world analysis confirms that AE reports for PPS exhibit a distinct long-latency risk profile. This is most obvious in the development of vision-threatening maculopathy, for which the mean time-to-onset for most specific pathologies is greater than 7 years. The analysis also identifies potential new safety signals, such as depression and anxiety, that are not currently reflected in the official drug labeling, and demonstrates distinct gender-specific risk profiles, with maculopathy signals being most prominent in females. These findings strongly underscore the necessity for establishing vigilant, long-term ophthalmologic screening protocols and broader safety monitoring for all patients receiving PPS therapy.

## Data Availability

The datasets presented in this study can be found in online repositories. The names of the repository/repositories and accession number(s) can be found at: https://fis.fda.gov/extensions/FPD-QDE-FAERS/FPD-QDE-FAERS.html.

## Footnotes

1
https://fis.fda.gov/extensions/FPD-QDE-FAERS/FPD-QDE-FAERS.html

2
https://www.fda.gov/drugsatfda
