# Correction: Post-marketing safety of pentosan polysulfate sodium: a 21-year pharmacovigilance analysis of the FAERS database

**DOI:** 10.3389/fmed.2026.1811896

**Published:** 2026-02-25

**Authors:** Ben Wang, Long Xia, Er-hao Bao, Yong-bo Zheng, Yu-han Li, Dan Han, Tian-yi Shao, Xian-zhi Liu, Ping-yu Zhu

**Affiliations:** 1 Department of Urology, Affiliated Hospital of North Sichuan Medical College, Nanchong, Sichuan, China; 2 Department of Urology, Sichuan Provincial People's Hospital East Sichuan Hospital & Dazhou First People's Hospital, Dazhou, China; 3 Department of Ultrasound, Affiliated Hospital of North Sichuan Medical College, Nanchong, China

**Keywords:** adverse drug events, FAERS database, maculopathy, pentosan polysulfate sodium, pharmacovigilance

In the published article, there was an error in the affiliation(s) for Tian-yi Shao, Xian-zhi Liu, and Ping-yu Zhu. Instead of “Department of Urology, Affiliated Hospital of North Sichuan Medical College, Nanchong, Sichuan, China”, it was erroneously given as “Department of Urology, Sichuan Provincial People's Hospital East Sichuan Hospital & Dazhou First People's Hospital, Dazhou, China.”

The **Author contributions** statement was erroneously given as [BW: Visualization, Methodology, Conceptualization, Writing – review & editing. LX: Formal analysis, Writing – review & editing, Data curation. E-hB: Methodology, Investigation, Writing – review & editing. Y-bZ: Writing – review & editing, Investigation, Validation. Y-hL: Software, Writing – review & editing. DH: Writing – original draft, Visualization. T-yS: Investigation, Writing – review & editing. X-zL: Writing – review & editing, Resources. P-yZ: Writing – review & editing, Project administration, Supervision]. The correct **Author contributions** statement is [BW: Conceptualization, Methodology, Visualization, Writing – original draft. LX: Data curation, Formal analysis, Writing – original draft. E-hB: Investigation, Methodology, Writing – original draft. Y-bZ: Investigation, Validation, Writing – original draft. Y-hL: Software, Writing – original draft. DH: Visualization, Writing – original draft. T-yS: Investigation, Writing – original draft. X-zL: Resources, Writing – original draft. P-yZ: Project administration, Supervision, Writing – review & editing].

The original version of this article has been updated.

